# Birds of a feather age together: telomere dynamics and social behavior predict life span in female Japanese quail *(Coturnix japonica)*

**DOI:** 10.3389/fendo.2024.1363468

**Published:** 2024-05-14

**Authors:** Shannon E. McCollum, Olivia Canter, Vincent J. Fasanello, Sarah Gronsky, Mark F. Haussmann

**Affiliations:** ^1^ Department of Biology, Bucknell University, Lewisburg, PA, United States; ^2^ Cellular and Molecular Biology, Michigan Medicine, University of Michigan, Ann Arbor, MI, United States; ^3^ Department of Chemistry, Duke University, Durham, NC, United States

**Keywords:** social support, telomere length, lifespan, social stress, aggression, anti-social behavior

## Abstract

Social support is vital for mental and physical health and is linked to lower rates of disease and early mortality. Conversely, anti-social behavior can increase mortality risks, both for the initiator and target of the behavior. Chronic stress, which also can increase mortality, may serve as an important link between social behavior and healthy lifespan. There is a growing body of literature in both humans, and model organisms, that chronic social stress can result in more rapid telomere shortening, a measure of biological aging. Here we examine the role of anti-social behavior and social support on physiological markers of stress and aging in the social Japanese quail, *Coturnix Japonica*. Birds were maintained in groups for their entire lifespan, and longitudinal measures of antisocial behavior (aggressive agonistic behavior), social support (affiliative behavior), baseline corticosterone, change in telomere length, and lifespan were measured. We found quail in affiliative relationships both committed less and were the targets of less aggression compared to birds who were not in these relationships. In addition, birds displaying affiliative behavior had longer telomeres, and longer lifespans. Our work suggests a novel pathway by which social support may buffer against damage at the cellular level resulting in telomere protection and subsequent longer lifespans.

## 1 Introduction

Social relationships are one of the most well-documented psychosocial factors that can affect a person’s mental and physical health (1, 2). High levels of social support correlate with lower rates of cardiovascular disease, cancer, and infectious disease (3, 4), while low social support correlates with increased morbidity and mortality (5). Conversely, the lack of social support (6), and more importantly antisocial behavior during childhood or adolescence is associated with higher rates of death and disability by middle age (7–9). Anti-social behavior describes acts that violate societal rules and norms such as theft or violence, often marked by aggression toward others. Interestingly, social support systems allow individuals to cope with stressful anti-social encounters (10). However, while the literature is rife with studies demonstrating the connection between social support and healthy aging, fewer studies explore how antisocial behavior affects health and aging (but see 8, 11), specifically at the cellular level.

Social stressors can trigger the highly-conserved vertebrate endocrine stress response, which can help individuals respond to challenges, such as an aggressive encounter (12). This multifaceted response is characterized by the release of catecholamines and glucocorticoids. Catecholamines result in rapid changes that define the fight or flight response, including increasing heart rate, altering blood flow, and mobilizing energy resources. Glucocorticoids work in tandem with catecholamines by modulating their effects, inhibiting unnecessary physiological functions, and assisting in energy mobilization that can help the animal to recover from the stress (13). Together, the actions of these hormones help individuals survive short-term stressors, but prolonged exposure to glucocorticoids is associated with increased levels of oxidative stress (14), cardiovascular disease (15), depression (16), and mortality risk (17).

At the cellular level, one way that prolonged glucocorticoid exposure may have negative health effects is by increasing oxidative stress (14, 18–21). Oxidative stress occurs when reactive oxygen species (ROS), many of which are byproducts of inefficient mitochondrial metabolism, damage biomolecules like DNA, lipids, and proteins (22). One molecule damaged by oxidative stress is telomeres (23), the terminal DNA caps important for chromosomal integrity. This damage, in addition to replication restrictions of DNA polymerase, result in progressive telomere shortening throughout both the life of the cell (24), and more broadly, the life of an organism (25). However, rather than just shortening due to the passage of time, telomeres may also shorten more rapidly, or slowly, based on the level of damage a cell is exposed to. Thus, telomeres are also a biomarker of biological aging (25, 26). Importantly, when telomeres shorten to a critical length, cells enter senescence: an irreversible change in which the cell ceases to divide and damage neighboring healthy cells. These changes result in a loss of the regenerative capacity of tissues and an increased risk of disease (27).

In social species, the complex social structure can either be a source of, or buffer against many types of stressors (28–30). In animals that live in groups, social interactions shape the relationships among individuals. Affiliative interactions strengthen social bonds, and provide benefits, while agonistic interactions are associated with costs that negatively affect social bonds (31). Individuals that are the continuous target of agonistic interactions can lead to long-term activation of the endocrine stress response and the subsequent negative health outcomes (28). Conversely, affiliative interactions that lead to membership in a social group can reduce exposure to the anti-social behavior, and thus lessen prolonged activation of the stress response (22). A recent review in humans, reported that chronic social stress, in both children and adults, is linked to telomere shortening (32). All of the studies reviewed were either cross-sectional or only followed subjects for only a short time as human longitudinal studies are difficult and very rarely follow subjects over their full lifetime. In this regard, social animal models can provide an incredibly useful framework for studies on lifespan. For example, experimental work in social birds showed that both pre- and post-natal stress can permanently after the sensitivity of the stress endocrine axis, which can affect longevity (33).

In this study, we explored the links between behavior, stress, and lifespan in the social Japanese quail (*Coturnix japonica*). Japanese quail are ideal models as they live in social groups and form dominance hierarchies, those these hierarchies are notably unstable and change through time depending on resource availability, season, and dynamic group relationships (34). While these quail show clear agonistic interactions–pecking and chasing–in the formation of these hierarchies, they also show affiliative behavior where they preferentially spend time with members of the group without engaging in agonistic interactions (35). We capitalized on this complex social system to explore how both affiliative and agonistic behavior affected baseline glucocorticoids, telomere rates of change, and lifespan.

## 2 Methods

All procedures were conducted with approval from the Bucknell University Institutional Animal Care and Use Committee and the reporting.

### 2.1 Study species

**Figure 1:**
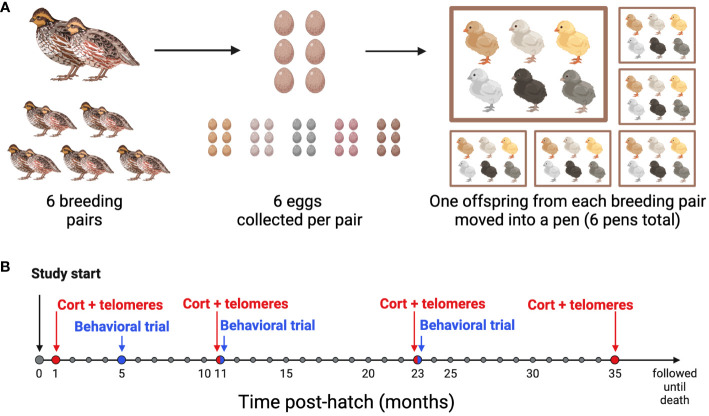
Experimental design timeline. **(A)** Breeding Schematic: 6 breeding pairs were age-matched, and 6 eggs were collected from each pair. At 4 weeks post-hatch, one offspring from each of the 6 families was moved into one of 6 pens, so that each pen had 6 unrelated offspring housed together. **(B)** Sampling and behavioral trial timeline: All birds were studied from hatch until death. Blood samples for baseline conrticosterone concentrations and telomere length (CORT + telomeres) were collected at 1, 11, 23, and 35 months of age. Behavioral Trials were performed at 5, 11, and 23 months of age. Created with BioRender.com.

A breeding colony of Japanese quail (*Coturnix japonica*) from a feral line originally captured on the Big Island of Hawaii in 1980 was maintained at Bucknell University as previously described (14). Because the birds were wild until recently, they have faced different natural selection pressures and have undergone less intensive artificial selection than domestic breeds of *Coturnix japonica* (36). In our colony, the birds have a mean lifespan 2.1 ± 1.08 (sd) years. The birds were housed in a 1.2 x 1.2 m pens and maintained on a light-dark cycle that mimicked the outdoor ambient light cycle in Lewisburg, Pennsylvania. Throughout the entire study, all birds received *ad libitum* access to food (Sporting Bird Starter and Sporting Bird Flight Developer, Southern States, USA) and water. For this study, six age-matched breeding pairs were established (180 ± 10 days) from the larger colony to produce six sibling sets each containing six genetically-related offspring (**Figure 1A**). Each parental pair lived in a separate pen for a period of two months at a 10-hour light:14 hour dark (10L:14D) cycle. To stimulate breeding, an additional hour of light was added to the start of the light cycle each week until a light cycle of 16L:8D was reached. Egg laying began sporadically when the light cycle reached 14L:10D, and we began collecting freshly laid eggs from each pair once the light cycle reached 16L:8D.

Within 24 hours of laying, eggs were placed in a GQF 1502 Digital Sportsman incubator (GQF Manufacturing Co., Savannah, GA, USA) maintained at 37.8°C and 50-65% humidity and were turned every 2 hours. On day 15 of incubation, the cohort was transferred to a GQF 1550 Digital Hatcher (1550 Cabinet Model, GQF Manufacturing Co., Savannah, GA, USA) and kept at 98°C and 85% humidity. Starting on day 18 of incubation, the eggs were checked every 12 hours for hatch. The 36 birds used in this study were all similarly aged and hatched within 9.1 ± 6.1 (sd) days of one another. After hatching, the birds were weighed, marked with numbered leg bands so we could track sibling sets, and housed in a Brower brooder maintained at 38°C. Temperature was then decreased at a rate of 2.5°C per week until 24°C was reached. At 14 days of age, we collected blood samples from the alar vein, and sex was determined via molecular sexing assay (see below). Only female offspring were used in this study. At approximately 4 weeks of age, offspring were transferred to six 1.2 × 1.2 m pens each housing six birds. Each pen contained one bird from each of the six sibling sets. Birds remained in their respective pens though the last sampling period.

### 2.2 Procedures

#### 2.2.1 Blood sampling for assays

Blood samples were taken at 14 days of age, and then again at one, eleven, twenty-three, and thirty-five months of age (**Figure 1B**). At each sample, approximately 75 µl of blood was collected from the alar vein within three minutes of entry into the room. Blood samples were collected with heparinized capillary tubes, transferred to microcentrifuge tubes, and kept on ice for a short period until refrigerated centrifugation (4°C) for 5 min at 3500 rpm. Plasma was removed and frozen in aliquots at -20°C. The remaining red blood cells were resuspended in a cryoprotectant buffer (10% dimethyl sulphoxide, 90% newborn bovine serum) and frozen at -20°C. All corticosterone and telomere assays were run at the end of the study after all samples were collected so that samples in each assay could be randomized by identity, family, pen, and age.

#### 2.2.2 Molecular sex determination

To identify the sex of each bird, we extracted genomic DNA from blood samples taken at two weeks of age using a modified version of the protocol developed by Griffiths et al. (37). The DNA was amplified using standard polymerase chain reaction (PCR) conditions with the P8⁄P2 primer set to determine the sex of each bird, based on the chromo-helicase-binding domain (CHD) gene in poultry (38). Amplified DNA was visualized after electrophoresis and sex was assigned based on the absence (male:ZZ) or presence (female:ZW) of the band for the W chromosome.

#### 2.2.3 Corticosterone RIA

We determined plasma corticosterone levels by radioimmunoassay (RIA), based on the protocol of Wingfield & Farner (39) and validated in our laboratory (14). Briefly, we extracted corticosterone from plasma diluted with double deionized water using anhydrous diethyl ether. The samples were dried with nitrogen gas and then resuspended in 90% ethanol and stored at 4°C overnight. Samples were then centrifuged, and the supernatant was dried under nitrogen gas and resuspended in phosphate-buffered saline with gelatin (PBSg).

We ran the samples in a competitive-binding RIA with a corticosterone-specific antibody Corticosterone-3-Carboxymethyloxime : BSAhost:rabbit (MP Biomedicals, Solon, OH, USA) and tritiated corticosterone (2000 cpm, NET 399, New England Nuclear Research Products, Boston, MA, USA). Bound and free corticosterone were separated using dextran coated charcoal. After centrifugation, charcoal was removed and radioactivity was determined using a liquid scintillation counter. Plasma CORT concentrations were determined from comparison against a standard curve. The average detection limit was approximately 0.30 ng/mL. All samples were run in duplicate, which were averaged for analysis, and the intra-assay and inter-assay coefficients of variation were 15.8% and 12.8%, respectively.

#### 2.2.4 Telomere length analysis

Telomeres were measured with the Telomere Restriction Fragment (TRF) assay, and the procedure was carried out according to previous studies (26, 40). Briefly, DNA was extracted from packed blood cells using the Puregene Blood Core Kit B following the manufacturer’s specifications (Qiagen). DNA integrity was assessed through the use of integrity gels (41), and telomeres of high integrity DNA samples were then measured using the TRF assay. A 10 μg quantity of DNA was digested using 1.0 ml of RsaI (New England Biolabs, R0167L) and 0.2 ml of HinfI (New England Biolabs, R0155M) in CutSmart Buffer (New England Biolabs, B7204S) overnight at 37°C. The digested DNA was separated using pulsed‐field gel electrophoresis (3 V/cm, 0.5‐ to 7.0‐s switch times, 14°C) for 19 hr on a 0.8% nondenaturing agarose gel. The gel was then dried without heating and hybridized overnight with a 32P‐labeled oligo (5′CCCTAA‐3′) that binds to the 3′ overhang of telomeres. Hybridized gels were placed on a phosphor screen (Amersham Biosciences, Buckinghamshire, UK), which was scanned on a Storm 540 Variable Mode Imager (Amersham Biosciences). We used densitometry (ImageQuant 5.03v and ImageJ 1.42q) to determine the position and strength of the radioactive signal in each of the lanes compared to the molecular marker (1 kb DNA Extension Ladder; Invitrogen, CA). The background was fixed as the nadir of the low‐molecular weight (MW) region on the gel (<1 kb). Samples were distributed among six gels and mixed by pen and age class.

#### 2.2.5 Behavioral trials

Behavioral observations to determine agonistic and affiliative interactions were conducted in each pen, during two one-hour trials at five, eleven, and twenty-three months of age (**Figure 1B**). A Canon Vixia HFS30s camera was suspended from the top of each pen four feet from the ground which could view the entirety of the pen floor. At the end of the light period on the day before the behavioral trial all food was removed from the pens so that the birds did not have access to food on the trial day at the start of the light period. On the trial day at 1300 hr, all birds were removed from the pen and temporarily placed into an adjacent pen. Birds were quickly placed back into the original pen one at a time and their band numbers were recited so that each bird could be identified when the video was later analyzed. This procedure of removing the birds and replacing them took less than two minutes. Following this, the birds were not disturbed for an additional 30 min. After this time, the food was replaced in the pen at ~1330 hr and interactions in the pen were recorded for the next 60 min. During the trials birds were very active as they were either attempting to access food, involved in agonistic behaviors (chases and pecking), or huddling together (affiliative behavior). This was repeated one week later at each of the three ages.

#### 2.2.6 Behavioral analysis

**Figure 2:**
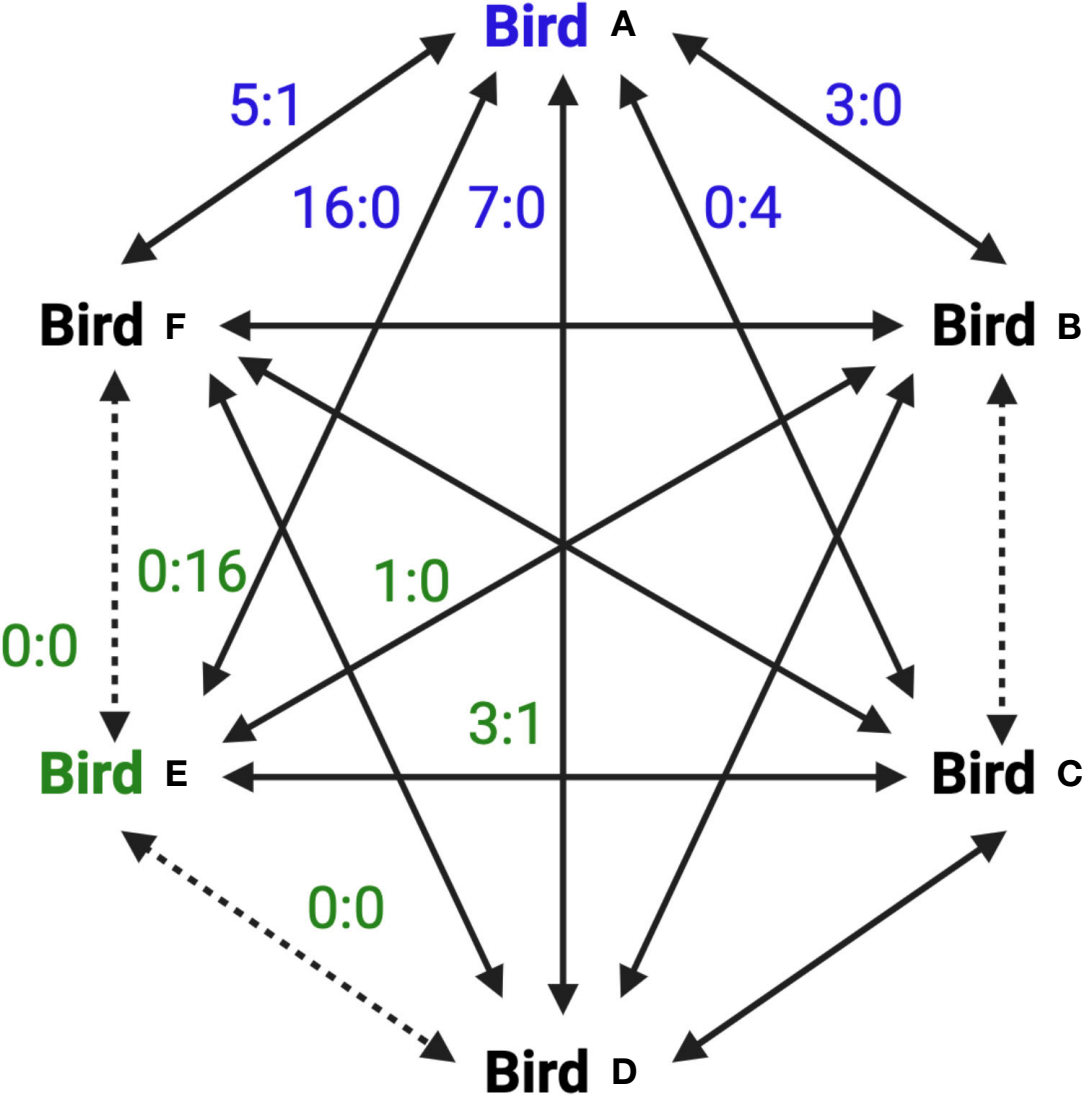
A behavioral trial diagram to illustrate the complex social relationships among birds in a pen. This diagram represents two birds in the pen and their interactions with their pen mates. Solid arrows denote when aggressive agonistic behaviors occurred between two pen members. Dashed arrows show where no aggressive agonistic behaviors occurred, which were categorized as affiliative relationships. Numbers beside arrows describe the nature of the interactions. The number before the colon is the number of aggressive actions (chases or pecks) that the focal bird performed on the target connected by the arrow, while the number after the colon is the numbers of times the focal bird was targeted by the bird connected by the arrow. For example, Bird A was involved in aggressive interactions with every other bird in the pen. It aggressed Bird F 5 times, and was targeted by Bird F once. Bird E was in two affiliative relationships, one with Bird F and the other with Bird D. While Bird A aggressed upon Bird E 16 times, Bird E never aggressed upon Bird A. This diagram displays data for Birds A and E for one of the two trials that occurred at a single age. While this diagram shows affiliative behavior between a number of Birds in the pen, this behavior would have to occurred in both the trials that were one week apart for these birds to actually be categorized in an affiliative relationship.

Videos were analyzed by two independent observers using Observational Data Recorder v2 Beta (Samuel Péan) and the data were averaged between both observations (repeatability calculated by the intraclass correlation coefficient = 0.97). This software allowed for easy observation of each bird over the entirety of the trial. We defined pecks and chases as agonistic interactions, and for each interaction we recorded which bird was the aggressor and which was the target. This also allowed us to determine when birds did not agonistically interact, which we defined as affiliative behavior (described below). A figure showing two birds from one pen, at one age, and for one of the two trials is shown to illustrate these complex relationships (**Figure 2**). Based off of these data we calculated four different behavioral scores.

• Agonistic aggressor score: To calculate agonistic aggressor score we divided the total times a quail aggressed other quail in their pen (chased or pecked others) by the total number of aggressive pen interactions among all quail in that pen for that trial.
• Agonistic target score: To calculate agonistic target score we divided the total times a quail was aggressed by the other quail in their pen (chased or pecked by others) by the total number of aggressive interactions among all quail in that pen for that trial.
• David’s Dominance Score: We used David’s score as a calculation of dominance behavior (42). David’s score is dependent on the outcomes of aggressive interactions with group members, as well as the relative strengths of each opponent. It also takes into consideration repeated interactions between group members and is therefore, not disproportionately impacted by slight deviations from the leading dominance direction. In addition, an individual’s status is independent of interactions in which they did not take part (42).

Affiliative score: Affiliative behavior in birds is complex, and can occur between both the same and opposite sexes (31, 35). A common measure of affiliative behavior is when specific members of a group spend time in close proximity without interacting agonistically (35, 43). During these affiliations birds may groom one another or rest in contact (31, 44). To measure affiliative behavior during the behavioral trials, if two or more birds rarely, or never, aggressively interacted they were categorized as displaying affiliative behavior. This measure of affiliative behavior, focusing on birds that huddled together rather than those engaged in resting or grooming behavior, was necessary. Resting and grooming were common behaviors observed in each pen outside of the behavioral trials. However, during the trials, the environment was more chaotic, with birds primarily engaged in eating, chasing and pecking (agonistic behaviors), or huddling together (affiliative behavior), and resting and grooming were not commonly observed. Affiliative behavior was analyzed by observing dyadic relationships, and this data allowed us to categorize each bird as having zero, one, or two affiliative relationships with other members of the pen (see **Figure 2** for an example) Specifically, for every pair combination in a pen, total aggressive pair encounters (each time those two individuals were involved in a chase or peck event) were divided by the total number of aggressive interactions among all quail in that pen for that behavioral trial. If this number of encounters was lower than 2%, those birds were considered to display affiliative behavior with one another. We chose this threshold because while analyzing behavior we noticed that on some rare occasions a chased bird may run into the back of another bird which might then quickly turn and peck it. This rare and undirected aggression, suggested we should slightly loosen our measure of affiliative behavior. In addition, we were only able to determine a bird’s affiliative membership status if we performed at least one behavioral analysis before they died. One bird died within the first year of life before the first behavioral trial, and so they were not included in any of the behavioral analyses.

### 2.3 Statistics

We ran linear mixed effects models (LMMs) or general linear mixed models (GLMMs) using JMP Pro software (v.14.0.0, SAS Institute Inc. 2018, Cary, NC, USA). For every LMM, we checked for homogeneity of variances (Levene’s test), and for normality of residuals (Kolmogorov–Smirnov test). If heteroscedasticity or non-normality were detected we switched to a general linear mixed model (GLMM) framework, as GLMMs can more easily accommodate non-normal and heteroscedastic data. For LMMs, we fitted the models with a Gaussian error distribution and an identity link function. For GLMMs we fitted the models with a gamma distribution and bounded variance components. Regardless of whether LMMs or GLMMs were used, in each model we included ‘individual’ as a random factor to control for the non-independence of data due to repeated measurements on the same individuals. ‘Pen’ (six pens) and ‘family’ (six families) were also included unless specified differently below. For all models that included interactions. we sequentially removed non-significant interactions from the models, starting from the higher order interactions, and repeated the analyses until we obtained a model with only significant terms.

#### 2.3.1 Behavioral trial repeatability analysis

Behavior repeatabilities of agonistic aggressor, agonistic target, and David’s score were calculated using the within- and between-variance components in a linear mixed effects model, following the restricted maximum-likelihood method (45, 46).

#### 2.3.2 Aggressive agonistic behavioral analysis

To determine what factors affected aggressive agonistic scores; we ran a LMM that included the fixed factors: affiliative behavior, age (four ages), corticosterone and all interactions.

#### 2.3.3 Affiliative behavioral analysis

To determine what factors affected whether birds were in an affiliative relationship, we ran a GLMM with a binomial response variable and logit link function with pen, family, corticosterone and all interactions included as model effects. Likelihood ratio tests following a chi-square distribution was used to assess significance of model effects.

#### 2.3.4 Telomere length analysis

To explore effects on telomere length, we ran analyses both on telomere length at four ages (1, 11, 23, and 35 months) and also on telomere loss rates expressed as change per month over the first and second year of the study. We did not include loss rates over the third year as 75% of the birds were dead by 35 months of age. In a first analysis, we ran a LMM and included age (four ages), CORT, and their interaction as fixed effects to see how they affected telomere length. In a second analysis, we ran a GLMM and included the four behavioral scores (aggressor, target, David’s, affiliative) and all interactions as fixed effects to determine how they affected telomere length. We could only include telomere at 11 and 23 months of age, as these were the only two where the behavioral trials and blood samples matched. Finally, we ran a GLMM to explore how the four behavioral scores and all interactions affected telomere loss rates. For the first year of telomere loss we used the 11 month behavioral trial data, and for the second year of loss we used the 23 month behavioral trial data.

#### 2.3.5 Lifespan analysis

To understand how a number of factors in our experiment affected lifespan we performed two analyses. First, we ran a LMM that included the fixed factors: average corticosterone levels, telomere length at 1 month of age, telomere loss rates in the first and second year, affiliative relationship status, and average David’s score, aggressor score, and target score. Limited sample size did not allow us to run interactions in this analysis. We used the results of this and previous analysis to more deeply explore the connection among significant variables using a path analysis. Briefly, path analysis is an extension of multiple regression that allows us to consider more than one dependent variable at a time and, more importantly, allows variables to be both dependent and independent variables (47, 48). In other words, it permits us to consider chains of association, where, for example, variable A influences variable B, which in turn affects variable C. For lifespan path analyses; affiliative relationship status and telomere loss rates (two measurements, change over the first and second year) were included.

Following standard procedures, we first evaluated a full (complete) path model, and subsequently tested simpler models that were nested within this basic model (49). Then, evaluation of alternative models involved several approaches:

• χ2 goodness of fit test statistic, in which a smaller value indicates better consistency with observed data (50).
• Root Mean Square Error of Approximation (RMSEA), which estimates the amount by which estimated values differ from actual values, was also used for model comparison. Values over 0.10 are considered to be a bad fit, those less than 0.08 reflect a reasonable fit, and values less than 0.05 indicate a good fit (50).
• Bentler’s Comparative Fit Index (CFI) is an incremental fit index that compares the fit of a hypothesized model with that of an independent model, which has the worst fit (51). This is In contrast to RMSEA which is an absolute fit index, in that it assesses how far a hypothesized model is from a perfect model. In the past, a CFI above 0.90 was used as a cutoff for good fitting models, but more recent consensus suggests that this value should be increased to approximately above 0.95 (52).
•Corrected Akaike information criterion (AICc) distinguishes between models derived from the maximum likelihood with the most parsimonious model being associated with the smallest AICc value as long as it is more than two AICc units lower than other models. AICc is especially useful where the sample size is small relative to the number of parameters being estimated.

Together, these tests provide a rigorous assessment and comparison of different models. For brevity, only model results for the top three models are presented.

## 3 Results

### 3.1 Behavioral trial repeatabilities

In each behavioral trial across the four years of the study, we consistently observed agonistic interactions in all pens. Individual’s aggressor score and David’s score were repeatable showing that levels of aggression and dominance, respectively, were consistent in individuals over time (**Table 1**). In addition, aggressor score, target score, and David’s score were repeatable in pens suggesting that some pens had consistently higher levels of aggression over time (**Table 1**).

**Table 1:** Repeatability of behavior across the bird’s lifespan, and also across families, and pens over time.

Behavioral trait	Predictor variables	*R*	n(i), n(m)	P
	Birds	**0.47**	**36, 75**	**0.01**
Aggressor score	Family	0.17	6, 75	0.2
	Pen	**0.07**	**6, 75**	**<0.0001**
	Birds	0.29	36, 75	0.07
Target score	Family	0.17	6, 75	0.2
	Pen	**0.07**	**6, 75**	**0.0006**
	Birds	**0.53**	**36, 75**	**0.01**
David’s score	Family	0.16	6, 75	0.3
	Pen	**0.07**	**6, 75**	**<0.0001**

n(i) = number of individuals, n(m) = total number of measurements. Bold text shows significant repeatabilities.

### 3.2 Aggressive agonistic behaviors

**Figure 3:**
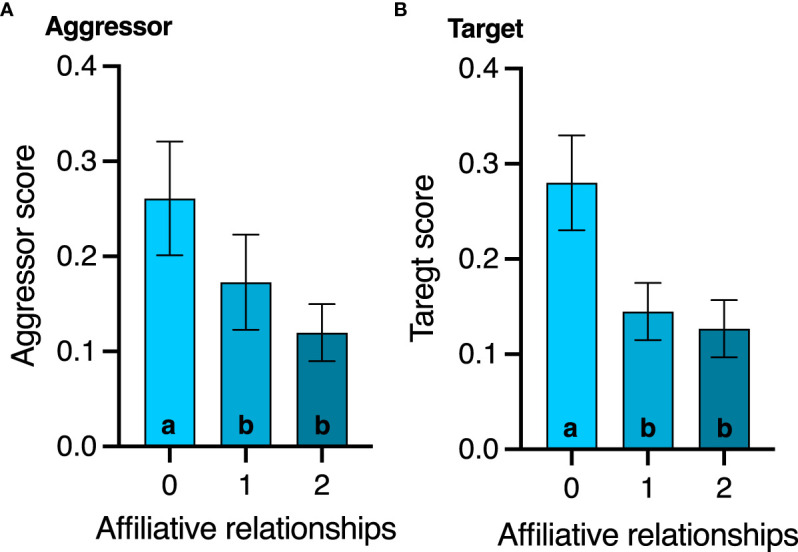
Effect of affiliative membership on **(A)** aggressor score and **(B)** victim score. Mean ± standard deviation is plotted. Different letters within a graph denote behavior score differences among the affiliative groups (p < 0.05).

None of the agonistic scores changed with age (all p>0.8, **Table 2**), but birds with the highest aggressor score also had significantly lower corticosterone levels regardless of age (F_1,17.1_ = 7.2; p=0.01, **Table 2**). While affiliative behavior had no effect on David’s score (p=0.5), it did have a significant effect on aggressor score (F_1,16.5_ = 4.4; p=0.05) and target score (F_1,32_ = 4.2; p=0.04, **Table 2**). Specifically, any birds that displayed affiliative behavior spent less time aggressing other birds (**Figure 3A**) and were also less likely to be a target (**Figure 3B**).

**Table 2:** Results of linear mixed models (LMM) analyzing the response to age, affiliative relationship, and corticosterone for the three aggressive agonistic behaviors.

Aggressive agonistic behavior	Age	Affiliative relationship	Corticosterone
Aggressor score	F_1,22_ = 0.01, p=0.8	**F_1,16.5_ = 4.4,** **p=0.05**	**F_1,17.1_ = 7.2,** **p=0.02**
Target score	F_1,32_ = 0.01, p=0.9	**F_1,32_ = 6.9,** **p=0.01**	F_1,32_ = 0.001, p=1.0
David’s score	F_1,32_ = 0.6, p=0.4	F_1,32_ = 0.2, p=0.9	F_1,32_ = 0.8, p=0.4

Bold values indicate p<0.05.

### 3.3 Affiliative behavior

Among our study birds, 14 (39%) did not display affiliative behavior, 12 (33%) had one single affiliative relationship, and 9 had two affiliative relationships (25%). Interestingly, affiliative relationships were remarkably stable over time, so any birds with an affiliative relationship that was found at the first behavioral analysis remained at the subsequent behavioral analyses as long as the birds in that relationship were still alive. Corticosterone levels (χ_1 _= 3.3; p=0.07), pen (χ_1 _= 3.3; p=0.07), and family (χ_1 _= 3.3; p=0.07) had no effect on whether birds were in affiliative relationships.

### 3.4 Telomere length

**Figure 4:**
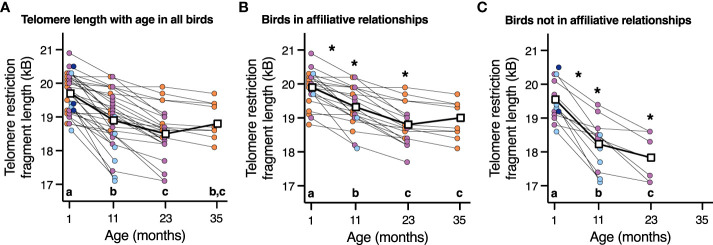
The relationship between telomere length and age in **(A)** all birds, **(B)** birds who are in an affiliation, and **(C)** birds who are not in an affiliation. Scatter plots are shown, and data points from individuals are joined by lines. For easier viewing of telomere length changes over time, birds are color-coded by approximate lifespan with birds who lived < 11 mo (dark blue), 11-23 mo (light blue), 23-35 mo (purple), and >35 mo (orange). Group means are denoted by open white squares joined by lines. Different letters within each plot denote telomere length differences among the ages on that specific plot (p < 0.05). An * denotes statistically significant difference among affiliative groups either at a specific age or rate of change shown in plot B and C at those specific ages (p < 0.05).

While there was no effect of corticosterone on telomere length (P=1.0), telomere length did decrease with advancing age (F_3,66.2_ = 57.7, P < 0.0001, **Figure 4A**). Aggressor score, target score, David’s score and all interactions were not associated with telomere length at any age or telomere loss rates (**Table 3**, all p>0.2). Telomere length decreased with age both in birds who were in affiliative relationships (F_3,42.3_ = 37.7, P < 0.0001, **Figure 4B**), and for birds who were not in affiliative relationships (F_2,21.4_ = 50.2, P < 0.0001, **Figure 2C**). However, birds displaying affiliative behavior had longer telomeres at both 11 and 23 mo (**Figures 4B, C**), and slower rates of telomere change over the first year of life, compared to birds that did not display affiliative behavior. Telomere length did not differ between these groups of birds at 1 mo of age (**Figures 4B, C**), and only birds who were in affiliative relationships were still alive at 35 mo of age (**Figures 4B, C**).

**Table 3:** Results of generalized linear mixed models (LMM) analyzing the response to aggressor score, target score, David’s score, and affiliative relationship on telomere lengths at different ages or telomere loss rates.

Telomere measurement	Aggressor score	Target score	David’s score	Affiliative membership
11-month length	F_1,20.8_ = 0.2, p=0.7	F_1,22.3_ = 3.4, p=0.08	F_1,20.1_ = 0.2, p=0.6	**F_1,23.1_ = 9.4,** **p=0.006**
23-month length	F_1,18.4_ = 1.2, p=0.3	F_1,9.5_ = 0.001, p=1.0	F_1,19.0_ = 0.8, p=0.4	**F_1,18.6 = _4.5,** **p=0.04**
First year, telomere loss per month	F_1,19.4_ = 1.3, p=0.3	F_1,14.3_ = 3.3, p=0.09	F_1,18.6_ = 0.002, p=1.0	**F_1,22.7_ = 12.9,** **p=0.002**
Second year, telomere loss per month	F_1,18.6_ = 0.5, p=0.5	F_1,16.2_ = 0.02, p=0.9	F_1,17.0_ = 1.3, p=0.3	F_1,18.5_ = 0.04, p=0.9

Bold values indicate p<0.05.

### 3.5 Lifespan

**Figure 5:**
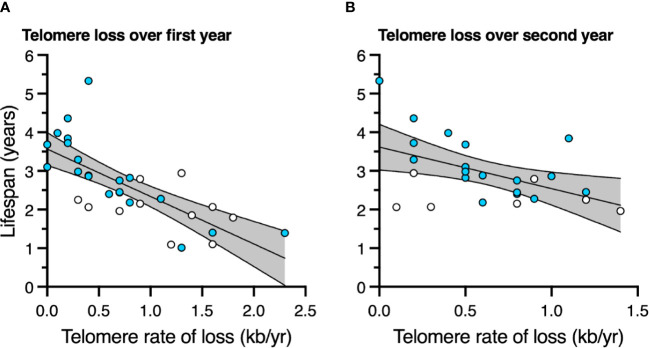
The relationship between telomere rate of loss and lifespan **(A)** over the first year of life, and **(B)** over the second year of life. Scatter plots are shown with data points for those birds not in an affiliation (white) and those in an affiliation (blue). Linear regression lines with 95% confidence intervals are plotted.

**Figure 6:**
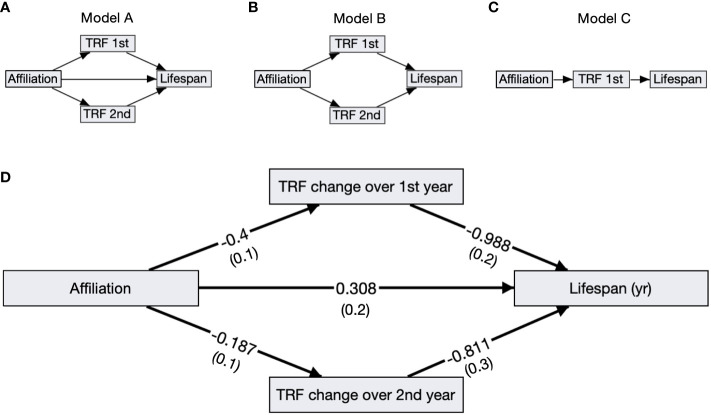
Path analysis diagrams. **(A–C)** Diagrams of the top ranked paths as determined by four model evaluation statistics; showing affiliative membership (Affiliation), telomere loss rates in the first (TRF 1st) and second year (TRF 2nd), and lifespan. **(D)** The top model A including standardized path coefficients with SEM in parentheses reported next to its corresponding arrow. All of the relationships, denoted by arrows, are significant in this model.

Birds with slower telomere loss rates in both the first and second year of life had significantly longer lifespans (**Table 4**, **Figure 5**). Average corticosterone concentrations, TRF length at 1 mo of age, and the four behavioral scores had no impact on lifespan (**Table 4**). Based on the significant relationships between affiliative behavior and telomeres and between telomeres and lifespan, we explored the linkages among these variables using path analysis. The top three models based on the four model evaluation statistics (**Table 5**) are shown **Figures 6A–C**. Importantly, all three of these models provided a good fit to the data and showed that affiliative relationships were related to telomere loss, which in turn predicted lifespan (**Figure 6**, **Table 5**). While distinguishing among these three models is difficult, for illustrative purposes, the model that satisfied all of the selection criteria (**Figure 6D**, **Table 5**) shows that for each for each affiliative relationship a bird possesses there was a reduction in telomere loss of 0.4kb in the first year (F_3,42.3_ = 37.7, P < 0.0001), and 0.2 kb in the second year (F_3,42.3_ = 37.7, P < 0.0001). In addition, each 1kb of telomere loss in the first year of life resulted in a 0.99 year reduction in lifespan, and in the second year of life, each 1kb of telomere loss subtracted an additional 0.82 years of life.

**Table 4:** Results of generalized linear mixed models (GLMM) analyzing the response average corticosterone concentration, TRF length at 1 mo of age, and TRF loss rates over the first and second year of life on lifespan.

Average corticosterone concentration	TRF length at 1 mo of age	TRF loss rate over first year	TRF loss rate over second year
F_1,6_ = 0.1, p=0.8	F_1,8.5_ = 0.7, p=0.4	**F_1,8.0_ = 6.7,** **p=0.03**	**F_1,6.1_ = 6.5,** **p=0.04**

Average David’s score	Average aggressor score	Average target score	Affiliative relationship status
F_1,6_ = 3.6, p=0.1	F_1,5.4_ = 1.4, p=0.3	F_1,7.8_ = 0.1, p=0.7	F_1,8.3_ = 0.3, p=0.6

Bold values indicate p<0.05.

**Table 5:** Model fit parameters for the null model and the three best-supported path analysis models affiliative membership, telomere loss in the first and second year, and lifespan.

Model	χ^2^	RMSEA	CFI	AICc
Null		0.47	0.00	285.12
Model A	**0.003**	**0.00**	**1.00**	**254.49**
Model B	**3.85**	0.16	**0.96**	**253.19**
Model C	9.02	0.24	0.87	**253.66**

Model generally fits to observed data if: 1) χ^2^ is not-significant, 2) RMSEA is ≤ 0.05; 3) CFI is > 0.95; and 4) the model has a small AIC value two units lower than other models. Bolded values in the table indicate that these criteria are satisfied.

## 4 Discussion

Our study investigates the effects of social support and anti-social behavior on chronic stress levels and lifespan in the social Japanese quail. We found that while affiliative behavior did not affect baseline corticosterone levels, it was linked to slower rates of telomere loss in the first and second year of life. In addition, affiliative behavior and telomere loss rates were independently connected to longer lifespans in these birds. To our knowledge, this is the first report of a connection between affiliative behavior, telomeres, and lifespan.

While agonistic interactions were common during behavioral trials, we also found that some pairs or trios of birds in each pen rarely or never engaged in aggressive encounters with one another. Importantly, these birds were still involved in aggressive encounters with other birds in the pen, but avoided aggressive interactions with one another. Affiliative behavior is common in social animals (53). In our study, these affiliative behaviors during the trials could provide a number of benefits including, but not limited to, (i) protecting members from agonistic interactions before they occur, so that less aggression is experienced, (ii) providing a social network to buffer against the negative effects of aggression after an encounter has occurred, or (iii) making an individual less likely to initiate agonistic interactions. We allowed social groups to form naturally, and therefore our experimental design does not allow us to differentiate among these three situations, which may not be mutually exclusive. Regardless, we found that birds displaying affiliative behavior were involved in less aggressive agonistic interactions throughout their lifespan. In humans, greater perceived social support is correlated with both less aggression toward others and less exposure to violence (54–56), suggesting that affiliative behavior could provide for both i and iii above. Interestingly, affiliative behavior may have a genetic component, as we found birds in some families were significantly more likely to be a part of a coalition. This is not entirely surprising, as genetic variation among individuals can lead to variation in social behavior and in turn, social information can alter brain gene expression and social behavior (57).

Birds in affiliative relationships had longer telomeres and less telomere loss over the first year of life compared to birds who did not display affiliative behavior. In humans, social support is also correlated with longer telomere length (58, 59 but see 60). Social support is known to decrease inflammation and oxidative stress (61, 62), both of which can shorten telomeres (23, 63). Thus, in our study, it is possible that affiliative behavior and the protection against agonistic interaction it provides may buffer cellular damage and protect telomeres.

Telomere length decreased with advancing age in the birds in our population. In addition, birds with slower telomere loss rates in both the first and second year of life had significantly longer lifespans. Previous work in birds (64–69) and humans (70–73) have reported that individuals with longer telomeres or slower loss rates have higher survival prospects (but see 74). To our knowledge, only one study to date has investigated telomere dynamics over the entire lifespan of the study organisms. Heidinger et al. (68) found that telomere length at 25 days of age in zebra finches was a very strong predictor of longevity. While we saw no effect of telomere length at one month of age on lifespan, we did find that telomere loss rates over the first year of life predicted lifespan. In agreement with Heidinger et al. (68), our work suggests that early life experiences and environment can have long-term effects on levels of cellular damage and organismal lifespan.

Due to the relationships between affiliative behavior, telomere loss, and lifespan, we performed path analysis to determine whether there was a causal pathway of effects between these three variables. The top three models were not easily distinguishable based on our four model selection criteria, and all show that affiliative relationships resulted in telomere loss in the first and second year of life compared to birds who were not involved in an affiliative relationship. In addition, birds with higher telomere loss rates also had shorter lifespans. Interestingly, in the model that passed all four selection criteria, affiliative behavior also had a beneficial effect on lifespan independent of its effects on telomeres. Other recent work in zebra finches showed that birds who experienced the most aggression had the highest levels of oxidative damage and the shortest telomeres (75). While this study did not measure affiliative behavior, it is possible that the birds that experienced the most aggression also received the smallest level of social support. Taken together with the work we present here, these results are exciting and suggest new possible lines of investigation for how social support, and specifically affiliative behavior could affect cellular aging and lifespan. We need more studies exploring the mechanistic links that underlie how social behavior can affect specific cellular damage and repair pathways.

One possible mechanistic link that connects social stressors to cellular damage are glucocorticoid levels. We measured baseline glucocorticoid levels at four times across the bird’s lifespans. Baseline glucocorticoids provide information on an individual’s energetic state, and baseline levels rise in response to frequent environmental challenges (18). We found that, regardless of age, birds with the highest agonistic aggressor score had significantly lower corticosterone levels. Higher levels of glucocorticoids inhibit aggressive behavior (76, 77), which agrees with the results from our study. In previous work from our lab, we found that being the target of aggressive behavior increased baseline corticosterone, but only if the individuals were exposed to high levels of maternal corticosterone during embryonic development (21). This previous study suggested that maternal glucocorticoids may provide for endocrine flexibility in stressful postnatal environments, and in the light of our current work it would be interesting to know if maternal glucocorticoid exposure may also prepare offspring for a stressful environment by increasing affiliative behavior.

While previous studies in humans (78–80) and birds (19, 81) reported links between glucocorticoids and telomere shortening; we did not find any connection between baseline corticosterone and telomeres in the present study. Circulating glucocorticoid levels are dynamic, and it is possible that simple measures of baseline levels do not fully capture this complexity. For example, previous work in chickens reported that telomere loss was not related to baseline levels of corticosterone, but rather corticosterone regulation through the efficacy of negative feedback (19). Interestingly, glucocorticoid regulation and the pattern of stress exposure also appears to be tied to increased oxidative stress (14). Ideally, future work would explore how lifetime measures of baseline and stress-induced glucocorticoid levels, as well as glucocorticoid negative feedback efficiency can affect cellular damage levels and organismal lifespan.

## 5 Conclusion

Social behavior is complex and consists of both beneficial affiliative behaviors and costly agonistic behaviors. In humans, high levels of social support are linked to better age-related outcomes, including reduced rates of cancer, increased immunity, and longer lifespan (3). Conversely, agonistic and antisocial behavior, which is difficult to study in humans, comes with several health risks (7). Using a model social animal, we found that affiliative behavior was linked with slower telomere attrition, and in turn, slower telomere attrition was linked with greater longevity. Our study highlights how the complexity of social behavior can have lifelong consequences. Future work should continue to address the underlying mechanisms by which social behavior affects aging and lifespan, and explore how environmental complexity and context can affect both affiliative and agonistic behaviors.

## Data availability statement

The raw data supporting the conclusions of this article will be made available by the authors, without undue reservation.

## Ethics statement

The animal study was approved by Bucknell Institutional Animal Care and Use Committee. The study was conducted in accordance with the local legislation and institutional requirements.

## Author contributions

SM: Validation, Visualization, Writing – original draft, Writing – review & editing. OC: Validation, Writing – original draft, Writing – review & editing. VF: Conceptualization, Data curation, Investigation, Methodology, Validation, Writing – original draft, Writing – review & editing. SG: Data curation, Investigation, Validation, Writing – original draft, Writing – review & editing. MH: Conceptualization, Data curation, Formal Analysis, Funding acquisition, Investigation, Methodology, Project administration, Resources, Software, Supervision, Validation, Visualization, Writing – original draft, Writing – review & editing.
